# Identification of optimal primary tumor resection candidates for metastatic gastric cancer: Nomograms based on propensity score matching

**DOI:** 10.1002/cam4.5983

**Published:** 2023-04-25

**Authors:** Zhehong Li, Honghong Zheng, Ziming Zhao, Guanyang Chen, Zheng Wang, Buhe Amin, Nengwei Zhang

**Affiliations:** ^1^ Department of General Surgery Beijing Shijitan Hospital, Capital Medical University Beijing China; ^2^ Department of Gastric Surgery Fujian Medical University Union Hospital Fuzhou China

**Keywords:** Metastatic gastric cancer, nomogram, primary tumor resection, propensity score matching, surgery, the SEER database

## Abstract

**Background:**

This study sought to develop and validate nomograms for screening patients with metastatic gastric cancer (mGC) who are candidates for primary tumor resection (PTR) and evaluating the prognosis of mGC patients after PTR.

**Methods:**

From 2010 to 2016, we screened mGC patients with complete data from the Surveillance, Epidemiology, and End Results (SEER) database. Depending on whether or not PTR was performed, we categorized patients into surgery and non‐surgery groups. A 1:1 propensity score matching (PSM) analysis was used to balance the characteristics of the two groups. The endpoints were overall survival (OS) and cancer‐specific survival (CSS). Two predictive nomograms were developed using logistic regression to assess the likelihood of benefit. Two additional prognostic nomograms were developed to assess prognosis in mGC patients after PTR by Cox regression. Finally, nomograms were evaluated using a variety of methodologies.

**Results:**

Our study included 3594 mGC patients who met the criteria. PTR was associated with improved OS and CSS time (median OS time after PSM: 15 vs. 7 months, *P* < 0.05; median CSS time after PSM: 17 vs. 7 months, *P* < 0.05). The OS‐related predictive nomogram, including age, histologic type, grade, T stage, and chemotherapy, was developed. Moreover, the CSS‐related predictive nomogram, including age, histologic type, grade, and chemotherapy, was developed. Sex, histologic type, grade, T stage, N stage, and chemotherapy were found to be correlated with OS. Furthermore, the CSS correlated with histologic type, grade, T stage, N stage, and chemotherapy. Both predictive and prognostic nomograms were found to be valuable and reliable after different types of validation.

**Conclusion:**

Predictive nomograms were developed and validated for identifying the optimal PTR mGC candidates. Prognostic nomograms were developed and validated for assessing the prognosis of mGC patients after PTR.

## Graphical abstract

Our study provides validated predictive and prognostic nomograms. Predictive nomograms assist surgeons in identifying metastatic gastric cancer patients who will benefit from primary tumor resection. Predictive nomograms can help surgeons evaluate the prognosis of metastatic gastric cancer patients after primary tumor resection. The novel models can help surgeons make better decisions and help specific patients.

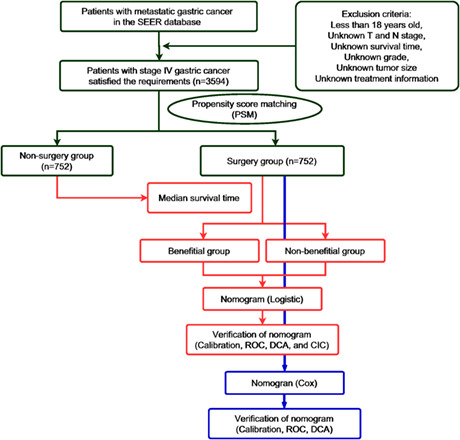

## 1 INTRODUCTION

Gastric cancer is the second most common cause of cancer deaths worldwide, with approximately 1,089,103 new cases and new 768,793 deaths in 2020.^1^, ^2^ Although early gastric cancer has a 5‐year survival rate of over 90%, more than 30% of patients are diagnosed with metastatic gastric cancer (mGC) at their first diagnosis.^3^, ^4^ Many studies have revealed that mGC individuals have a dismal prognosis, with a median survival time of <1 year and a 5‐year survival rate of fewer than 5%.^4^, ^5^, ^6^ MGC patients have a poor prognosis, so choosing the appropriate treatment following diagnosis is critical to enhancing their prognosis.

The combination of lymph node dissection and complete primary tumor resection (PTR) has improved the prognosis of early gastric cancer and non‐early operable gastric cancer patients.^7^ However, in mGC patients, PTR is not usually considered a standard treatment option.^8^ It is worth mentioning that PTR is not a radical procedure for mGC patients but rather a palliative one.^9^, ^10^ Symptom relief, illness control, and tumor load reduction can be achieved by PTR.^9^, ^11^ Retrospective studies by Choi et al. and Lin et al. supported the hypothesis that patients with recurrent or mGC can benefit from PTR and/or metastatic tumor resection.^12^, ^13^ Although PTR can assist mGC patients, Smith et al^14^ mentioned that mGC people having PTR are a highly select group. Lasithiotakis et al^15^ revealed that not all mGC patients are suited for PTR, and highly individualized patient profiles such as age, tumor status, and chemotherapy must be considered. These studies have suggested that PTR can improve the prognosis of some mGC patients, but the exact subset of individuals who benefit from the therapy is yet unknown.

As a result, in this study, we developed and validated novel diagnostic prediction models using a public database to identify mGC patients who could benefit from PTR. Novel prognostic prediction models for mGC patients were also developed to assess their prognosis after PTR.

## 2 METHODS

### 2.1 Patients

We have been granted access to The Surveillance, Epidemiology and End Results (SEER) database (15708‐Nov2020). During the study period from 2010 to 2016, the SEER stat program selected patients diagnosed with gastric cancer by histopathology from the SEER database (8.4.0). Also, the based clinicopathological characteristics (age, race, sex, histology type, tumor site, tumor size, grade, T stage, N stage, M stage, radiotherapy, chemotherapy, surgery, and follow‐up information) were collected. Patients diagnosed as mGC were enrolled. The criteria for exclusion were as follows: unknown T and N stage, unknown survival time, unknown grade, unknown tumor size, unknown therapy information, and under 18 years of age.

Overall survival (OS) was computed from the date of diagnosis to the date of death owing to any cause. Cancer‐specific survival (CSS) was defined as the time from the date of diagnosis to death due to gastric cancer.

### 2.2 Propensity score matching (PSM) and data processing

Whether or not PTR performed, eligible mGC patients were separated into surgery and non‐surgery groups. PSM analysis was used to reduce bias by equating the two groups based on the following variables: age, race, sex, tumor site, histologic type, grade, T stage, N stage, tumor size, radiotherapy, and chemotherapy. Individuals were subsequently matched using a 1:1 nearest‐neighbor matching algorithm with a caliper width of 0.05 SDs of the propensity score logit and without replacement as suggested by simulation studies. Kaplan–Meier (K‐M) analysis and log‐rank test were used to explore the difference in prognosis between the two groups before and after PSM. After PSM, patients in the surgery group were separated into training set (70%) and validation set (30%). Patients from the training set were used to develop the nomogram, and patients from the validation set were utilized to validate it in this study.

### 2.3 Construction and verification of nomogram

After PSM, mGC patients in the surgery group whose median survival time was longer than the median OS and CSS time in the non‐surgery group were regarded as the surgery‐beneficial group; otherwise, they were labeled as the surgery‐non‐beneficial group. The independent predictive factors linked to surgical benefit were determined using multivariate logistic analysis. The predictive nomograms were developed based on the independent predictive factors. At the same time, we generated the receiver operating characteristic (ROC) curve and evaluated the nomogram's discriminative capability using the area under the curve (AUC). In addition, comparable calibration curves were produced to demonstrate the nomogram's calibration performance, and decision curve analysis (DCA) and clinical impact curve (CIC) were used to demonstrate the clinical benefits of the nomogram. Multivariate Cox regression analysis determined independent prognostic factors in mGC patients after surgery. Based on the independent prognostic parameters, prognosis nomograms were developed. The two prognostic nomograms were also evaluated by ROC curves, calibration curves, and DCA.

### 2.4 Statistical analysis

The categorical variables were analyzed by chi‐square test. R software, version 4.1.1, was used for all statistical calculations. The survival analyses were performed by the K‐M method and the log‐rank test. All statistical tests were two‐sided, and *p* < 0.05 is considered statistically significant.

## 3 RESULTS

### 3.1 Flowchart

**FIGURE 1:**
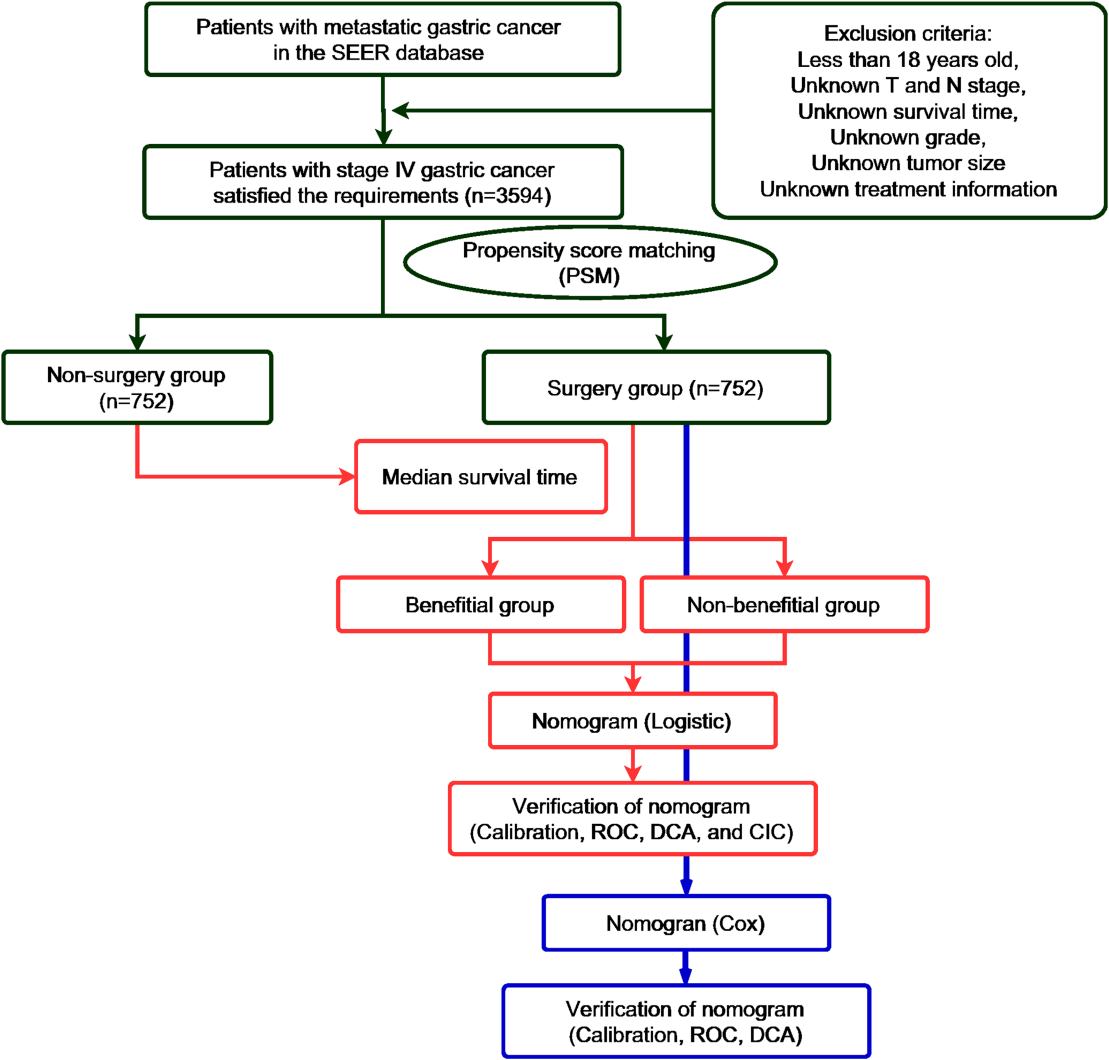
Flowchart of the study.

Figure 1 depicts a detailed workflow.

### 3.2 Patient characteristics before and after PSM

**FIGURE 2:**
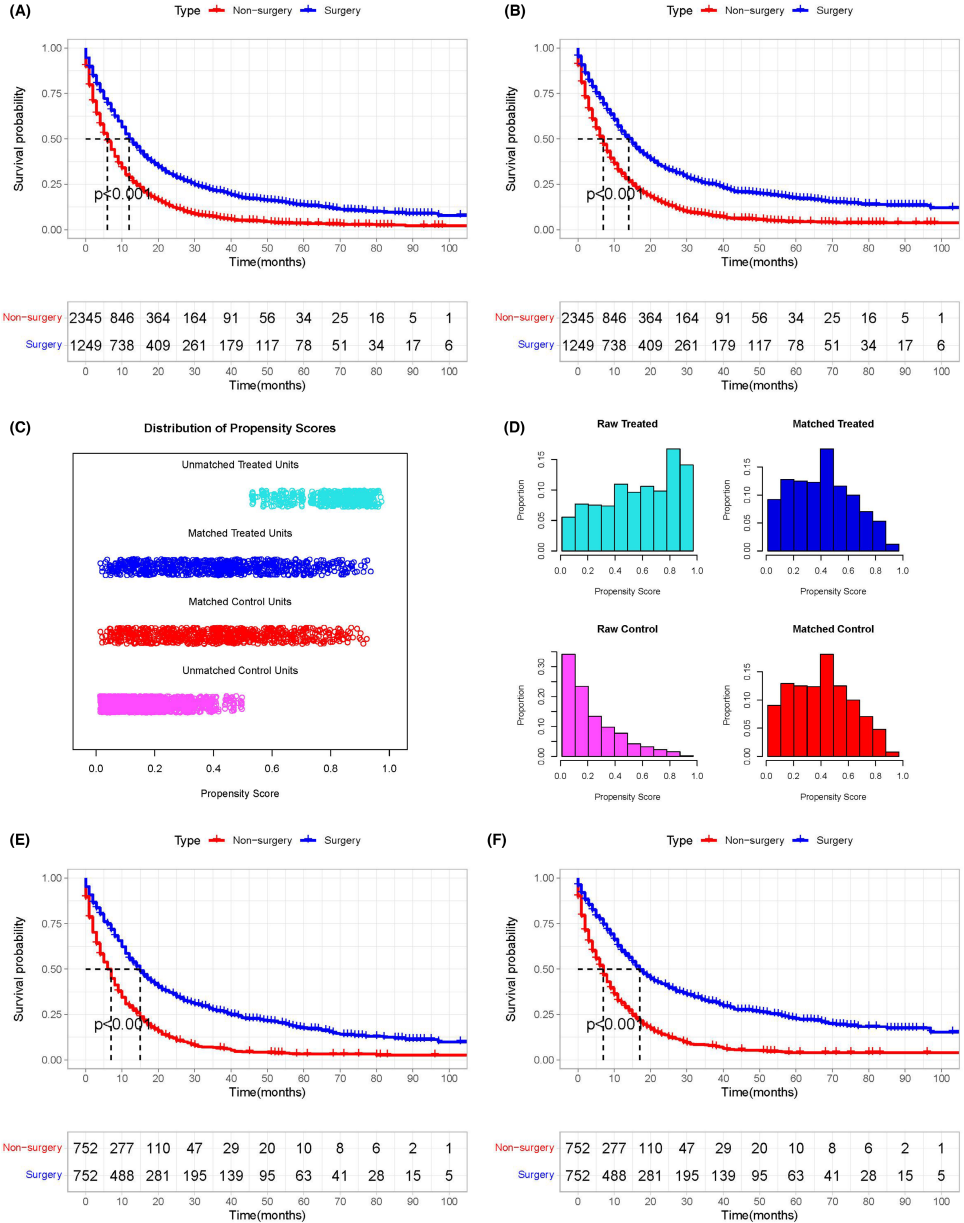
Kaplan–Meier plot and PSM analysis of mGC patients according to surgery. Before PSM, the surgery group had a better OS (A, 12 vs. 6 months, *p* < 0.001) and CSS (19 vs. 7 months; *p* < 0.001) than the non‐surgery group. Jitter plots (C) and histograms (D) showing PSM improved the balance of clinicopathological information between the surgery and non‐surgery groups. Kaplan–Meier plot and PSM analysis of mGC patients according to surgery. After PSM, the surgery group had a better OS (E, 15 vs. 7 months, *p* < 0.001) and CSS (16 vs. 7 months; *p* < 0.001) than the non‐surgery group. CSS, cancer‐specific survival; PSM, propensity score matching; mGC, metastatic gastric cancer; OS, overall survival.

The study included 3594 mGC patients who met the inclusion and exclusion criteria. The surgery group included 1249 mGC patients (34.75%) who had performed PTR, while the non‐surgical group included 2345 mGC patients (65.25%). In the K‐M method and log‐rank test (see Figure 1A,B) and subgroup analysis (see Figures S1 and S2), patients who underwent PTR had longer OS and CSS time. After 1:1 PSM, 1504 patients treated with or without PTR were enrolled in the following analysis (see Figure 2C,D and Table 1). After PSM, K‐M method and log‐rank test (see Figure 1E,F) and subgroup analysis (see Figures S3 and S4) also showed that patients who underwent primary tumor resection had longer OS and CSS time. Seven hundred fifty‐two mGC patients in the surgery group were randomly divided into the training set (*n* = 528) and the validation set (*n* = 224), and relevant patient data were shown in Table S1. The median OS and CSS time were 7 months. Therefore, we defined benefits for mGC patients who underwent PTR and had OS or CSS time for more than 7 months. Based on the benefits definition, 539 patients (OS time > 7 months) were assigned to the OS‐related surgery‐beneficial group and 565 patients (CSS time > 7 months) were assigned to the CSS‐related surgery‐beneficial group.

**TABLE 1:** Clinical characteristics of participants before and after PSM.

Variable	Before PSM		SMD	*p*	After PSM		SMD	*p*
	Non‐surgery group *N* = 2345 (%)	Surgery group *N* = 1249 (%)			Non‐surgery group *N* = 752 (%)	Surgery group *N* = 752 (%)		
Age (years)								
<65	710 (30.28)	515 (41.23	−0.084	<0.001	365 (48.54)	363 (48.27)	0.005	0.918
≥ 65	1635 (69.72)	734 (58.77)			387 (51.46)	389 (51.73)		
Gender								
Female	710 (30.28)	515 (41.23)	−0.223	<0.001	269 (35.77)	277 (36.84)	−0.022	0.668
Male	1635 (69.70)	734 (58.77)			483 (64.23)	475 (63.16)		
Race								
White	1765 (75.27)	790 (63.25)	0.249	<0.001	509 (67.69)	513 (68.22)	−0.020	0.841
Black	296 (12.62)	207 (16.57)			117 (15.56)	121 (16.09)		
Other	284 (12.11)	252 (20.18)			126 (16.76)	118 (15.69)		
Histologic type								
Adenocarcinoma (exclude signet ring cell)	1630 (69.51)	603 (48.28)	0.410	<0.001	429 (57.05)	421 (55.98)	0.030	0.781
Signet ring cell	330 (14.07)	259 (20.74)			125 (16.62)	121 (16.09)		
Other	385 (16.42)	387 (30.98)			198 (26.33)	210 (27.93)		
Tumor site								
Cardia and Fundus	1215 (51.81)	197 (15.77)	0.695	<0.001	258 (34.31)	184 (24.47)	−0.008	<0.001
Body	206 (8.78)	132 (10.57)			62 (8.24)	94 (12.50)		
Antrum and pylorus	293 (12.49)	428 (34.27)			117 (15.56)	241 (32.05)		
Lesser and greater	311 (13.26)	184 (14.73)			146 (19.41)	106 (14.10)		
Other	320 (16.65)	308 (24.66)			169 (22.47)	127 (16.89)		
Tumor size (cm)								
<3.0	612 (26.10)	177 (14.17)	0.391	<0.001	149 (19.81)	140 (18.62)	0.006	0.699
3.0–5.9	1024 (43.67)	498 (39.87)			289 (38.43)	304 (40.43)		
≥ 6.0	709 (30.23)	574 (45.96)			314 (41.76)	308 (40.96)		
Grade								
Grade I‐II	665 (28.36)	320 (25.62)	0.063	0.080	210 (27.93)	219 (29.12)	−0.027	0.607
Grade III‐IV	1680 (71.64)	929 (74.38)			542 (72.07)	533 (70.88)		
T stage								
T1	760 (32.41)	49 (3.92)	1.165	<0.001	86 (11.44)	48 (6.38)	0.017	<0.001
T2	192 (8.19)	67 (5.36)			43 (5.72)	65 (8.64)		
T3	704 (30.02)	394 (31.55)			246 (32.71)	306 (40.69)		
T4	689 (29.38)	739 (59.17)			377 (50.13)	333 (44.28)		
N stage								
N0	900 (38.38)	254 (20.34)	0.814	<0.001	170 (22.61)	238 (31.65)	−0.01	<0.001
N1	1089 (46.44)	258 (20.66)			344 (45.74)	225 (29.92)		
N2	217 (9.25)	248 (19.86)			121 (16.09)	164 (21.81)		
N3	139 (5.93)	489 (39.15)			117 (15.56)	125 (16.62)		
Radiotherapy								
No	1768 (75.39)	1076 (86.15)	−0.311	<0.001	603 (80.19)	617 (82.05)	−0.054	0.356
Yes	577 (24.61)	173 (13.85)			149 (19.81)	135 (17.95)		
Chemotherapy								
No	783 (33.39)	498	−0.132	<0.001	254 (33.78)	262 (34.84)	−0.022	0.516
Yes	1562 (66.61)	751			498 (66.22)	490 (65.16)		

Abbreviations: PSM, propensity score matching; SMD, standardized mean difference.

### 3.3 Development and validation of predictive models

**FIGURE 3:**
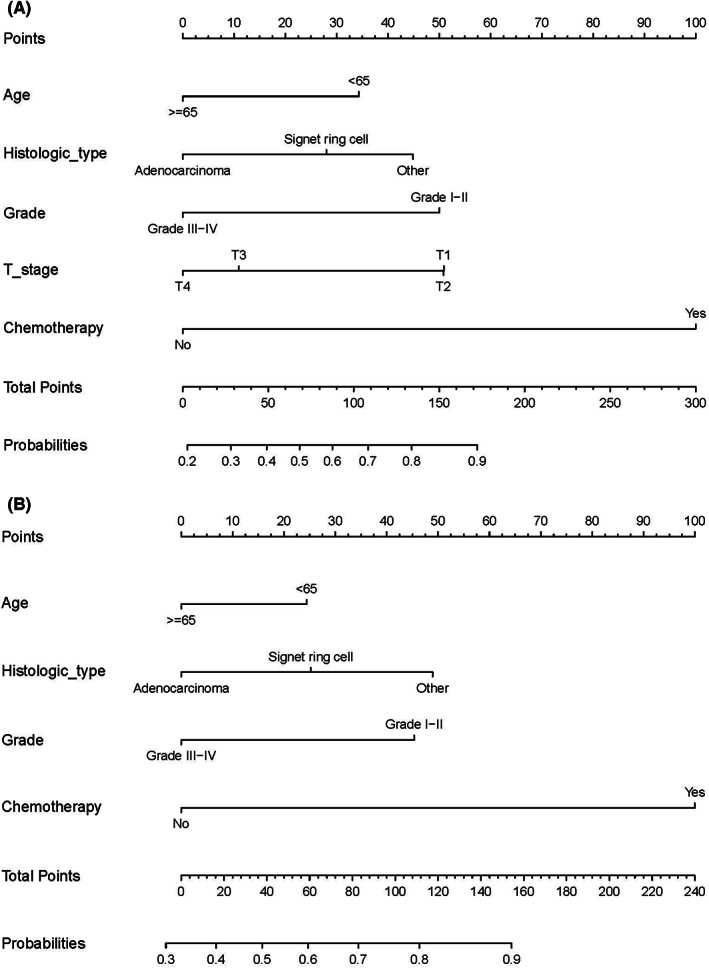
Predictive nomogram. Predictive nomogram to select optimal mGC patients who could gain OS (A) and CSS (B) benefits from surgery. CSS, cancer‐specific survival; OS, overall survival.

Independent predictive factors were derived by multivariate logistic analysis in the training set, including age, histologic type, grade, T stage, and chemotherapy in the OS‐related cohort (see Table S2). Moreover, age, histologic type, grade, and chemotherapy in the CSS‐related cohort (see Table S2). Based on the independent predictive factors, we build two predictive nomograms (see Figure 3).

There were two steps to using the nomogram. First, the score for each factor was determined by drawing a straight line from that factor upwards. Second, added all factor scores and constructed a straight line down to evaluate the likelihood of benefit following surgical resection of the primary site in mGC patients. For example, a 70‐year‐old mGC patient with a histological diagnosis of gastric adenocarcinoma (exclude signet ring cell), T3 stage, grade IV, had received chemotherapy and a total score of 111 from the OS‐related nomogram. In this particular instance, the nomogram projected that PTR would result in a 71% chance of benefit. Patients in the surgery‐beneficial group had a better than 50% chance of benefit, whereas those in the surgery‐non‐beneficial group a chance of benefit of less than or equal to 50 percent.

**FIGURE 4:**
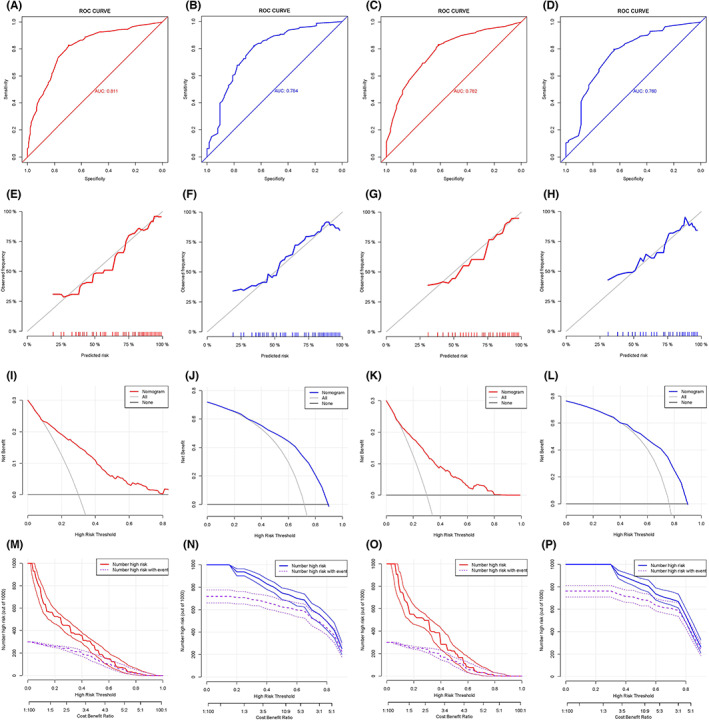
Multiple evaluation methodologies of predictive nomogram. ROC curves for predicting OS‐related surgery beneficial mGC patients in training set (A, AUC = 0.811) and validation set (B, AUC = 0.784). ROC curves for predicting CSS‐related surgery beneficial mGC patients in training set (C, AUC = 0.782) and validation set (D, AUC = 0.760). The calibration curves for predicting OS‐related surgery beneficial mGC patients in the training set (E) and validation set (F). Calibration curves for CSS‐related surgery beneficial mGC patients in the training set (G) and validation set (H). The DCA curve for predicting OS‐related surgery beneficial mGC patients in the training set (I) and validation set (J) and CSS‐related surgery beneficial mGC patients in the training set (K) and validation set (L). The CIC curve for predicting OS‐related surgery beneficial mGC patients in the training set (M) and validation set (N) and CSS‐related surgery beneficial mGC patients in the training set (O) and validation set (P). CIC, clinical impact curve; CSS, cancer‐specific survival; DCA, decision curve analysis; mGC, metastatic gastric cancer; OS, overall survival; ROC, receiver operating characteristic.

**FIGURE 5:**
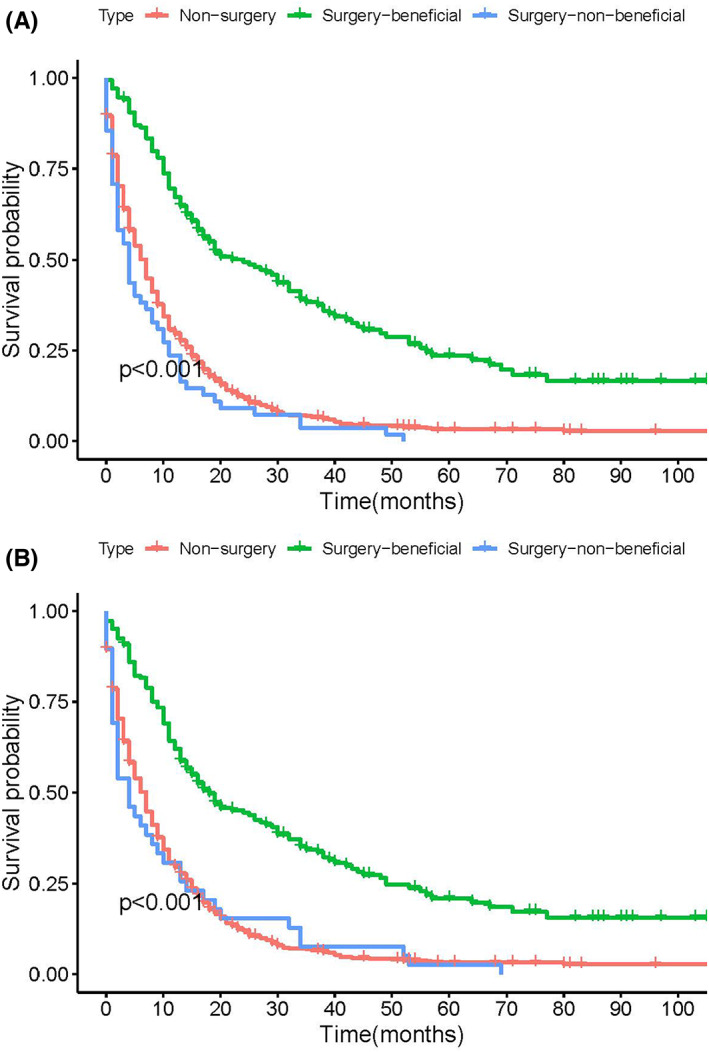
Kaplan–Meier plot. Kaplan–Meier plot to differentiate OS‐related (A) and CSS‐related (B) beneficial group according to the predictive nomogram after PSM in validation set. CSS, cancer‐specific survival; OS, overall survival; PSM, propensity score matching.

In the ROC curve analysis, the AUC of the nomograms (see Figure 4A–D) for OS‐ and CSS‐related optimal primary tumor resection candidates prediction was 0.811 and 0.782 in the training set and 0.784 and 0.760 in the validation set, respectively. The calibration curves also showed two nomograms with good calibration (see Figure 4E–H). The clinical effectiveness of two nomograms were confirmed by DCA and CIC (see Figure 4I–P). We then validated the distinguish ability of the two nomograms in the validation set by the K‐M method and the log‐rank test (see Figure 5A,B). The surgery‐beneficial group has significantly longer OS and CSS time than surgery‐non‐beneficial and non‐surgery group (*P* < 0.05), but no difference was observed between the surgery‐non‐beneficial and non‐surgery group (*P* > 0.05).

### 3.4 Development and validation of prognostic models

**FIGURE 6:**
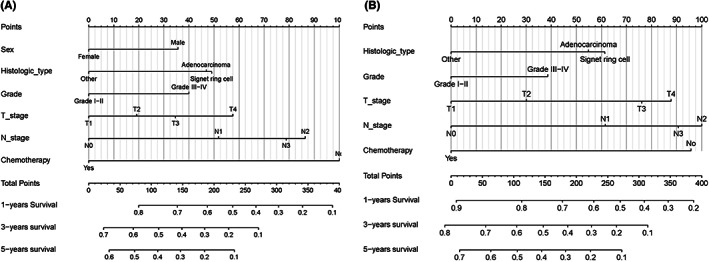
Prognostic nomogram. Nomograms predicting 1‐, 3‐, and 5‐year OS (A) and CSS (B). CSS, cancer‐specific survival; OS, overall survival.

**FIGURE 7:**
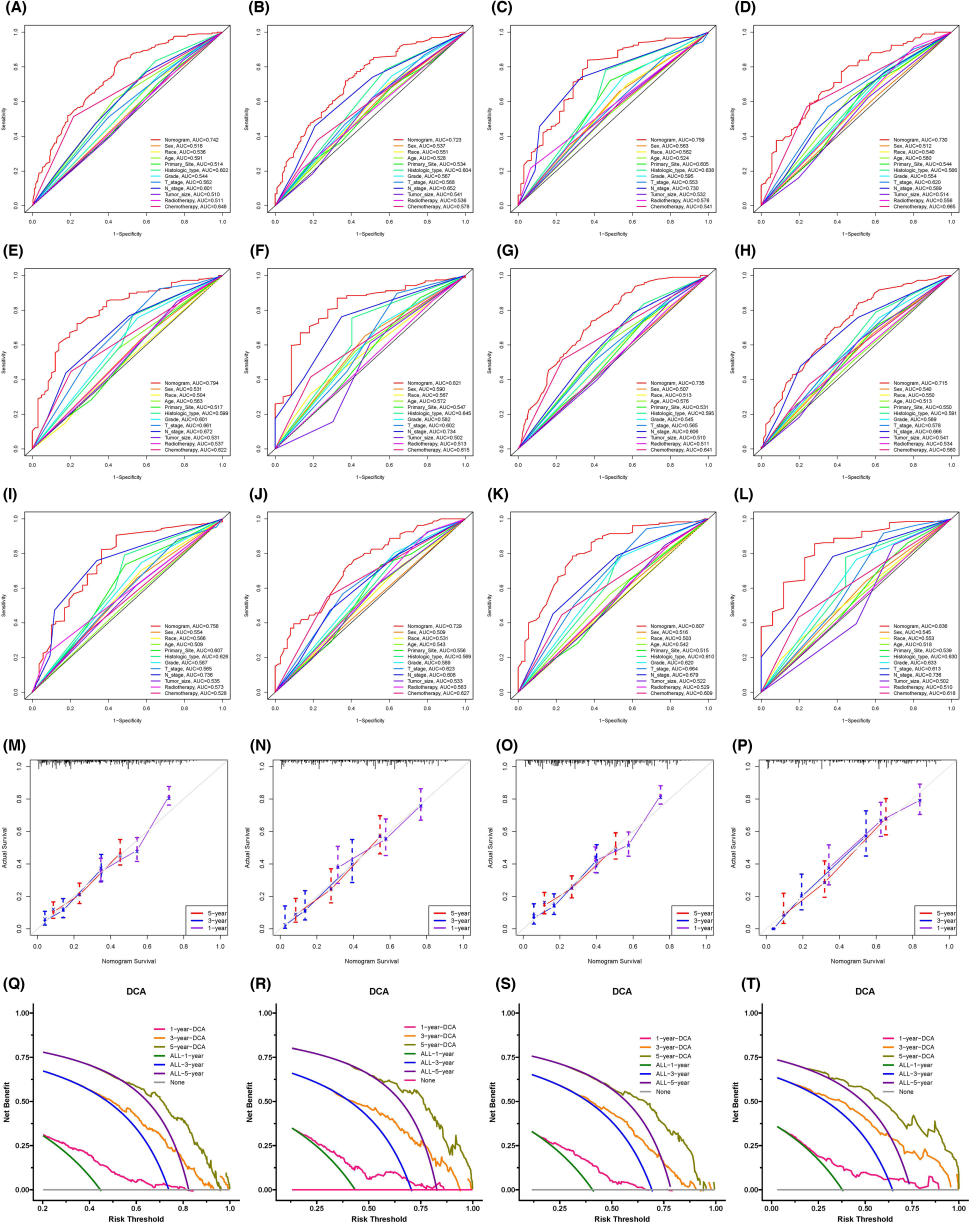
Multiple evaluation methodologies of prognostic nomogram. ROC curves for predicting 1‐year (A), 3‐year (B), and 5‐year (C) OS in the training set, and 1‐year (D), 3‐year (E), and 5‐year (F) OS in the validation set. ROC curves for predicting 1‐year (G), 3‐year (H), and 5‐year (I) CSS in the training set, and 1‐year (J), 3‐year (K), and 5‐year (L) CSS in the validation set. Calibration curves of the nomogram for the OS prediction in the training set (M) and validation set (N). Calibration curves of the nomogram for the CSS prediction in the training set (O) and validation set (P). DCA of the nomogram for the OS prediction in the training set (Q) and validation set (R). DCA of the nomogram for the CSS prediction in the training set (S) and validation set (T). CSS, cancer‐specific survival; DCA, decision curve analysis; OS, overall survival; ROC, receiver operating characteristic.

Independent prognostic factors were derived by multivariate Cox analysis in the training set, including sex, histologic type, grade, T stage, N stage, and chemotherapy in the OS‐related cohort (see Table S3). Moreover, independent prognostic factors, including histologic type, grade, T stage, N stage, and chemotherapy, were identified in the CSS‐related cohort (see Table S3). We built two prognostic nomograms based on the independent prognostic factors (see Figure 6). In the ROC curve analysis, the AUC of the nomogram for predicting 1‐, 3‐, and 5‐year OS was 0.742, 0.723, and 0.759 in the training set (see Figure 7A–C). In the validation set, the AUC of the nomogram for predicting 1‐, 3‐, and 5‐year OS was 0.730, 0.794, and 0.821 (see Figure 7D–F). We also found that the nomogram's discriminative power was likewise found to be superior to clinicopathological characteristics alone. AUC of the nomogram used to predict 1‐, 3‐, and 5‐year CSS showed better predictive value in both training and validation sets (see Figure 7G–L). The 1‐, 3‐, and 5‐year OS and CSS probability calibration curves also showed that the nomogram's results and actual outcome were in good agreement (see Figure 7M–P). The clinical effectiveness of 1‐, 3‐, and 5‐year OS and CSS probability were confirmed by DCA (see Figure 7Q–T).

## 4 DISCUSSION

This study was performed by stratifying four perspectives. Firstly, the conclusions of the SEER database analysis were consistent with the findings of other scholars: survival benefits of PTR for some mGC patients.^12^, ^13^, ^14^ Secondly, we developed two predictive nomograms for determining whether patients with mGC could benefit from PTR in this study. Thirdly, we also developed two novel prognostic nomograms to better understand the prognosis of mGC patients after PTR. Finally, internal validation proved that our models were both valuable and stable.

Gastric cancer is characterized by a high incidence and insidious onset, and in its early stages, there are no evident or usual clinical symptoms.^1^ Studies have shown that patients with mGC have a poor prognosis, with a median survival time of <1 year and a 5‐year survival rate of <5%.^4^, ^5^, ^6^ The surgical treatment of gastric cancer with R0 resection and related site lymph node dissection can result in a favorable prognosis for individuals in the early and progressive stages. However, it is still controversial whether to perform PTR for mGC patients. Schmidt et al^16^ concluded that the median OS of mGC patients in the surgery group was not statistically significant compared to the nonoperatively managed group (*p* > 0.05). Moreover, Schmidt et al found that noncurative resections (excluding emergencies) considerably increase perioperative mortality and morbidity.^17^ It is not a coincidence that Fujitani et al^17^ came to the conclusion that non‐curable surgery reduces chemotherapy compliance without providing any prognostic benefit. Another study has presented a contrasting viewpoint, proposing that palliative surgical resection gives stage IV gastric cancer patients with survival benefits.^18^, ^19^ Meanwhile, Sun et al^20^ also further confirmed that cancer‐directed surgery was beneficial to the prognosis of IV gastric cancer patients using SEER datasets. There are various reasons why people with mGC benefit from the PTR. Firstly, the risk of complications related to gastric cancer, such as bleeding, obstruction, and carcinoid syndrome, was relieved when PTR was performed. Secondly, PTR assisted in confirming the diagnosis and determining the optimal course of treatment. However, Lasithiotakis et al^15^ revealed that not all mGC patients are suited for PTR, and highly individualized patient profiles such as age, tumor status, and chemotherapy must be considered. In our study, the term “actual benefit” referred to postoperative mGC patients who underwent PTR and survived for a longer period of time than non‐surgery group for both OS and CSS. Our study showed that the tow predictive nomograms assisted surgeons in identifying mGC patients who will benefit from PTR.

The OS‐related predictive nomogram included age, histologic type, grade, T stage, and chemotherapy as relevant predictors. In the CSS‐related predictive nomogram, age, histologic type, grade, and chemotherapy were all significant predictors. Chemotherapy is the most common palliative treatment for people with mGC.^21^, ^22^ According to Sano et al^23^ research, after effective chemotherapy, PTR and/or metastatic tumor resection improve patient survival. Our research also emphasized the significance of chemotherapy. Our study showed that chemotherapy in the nomogram accounted for a large proportion in evaluating whether patients were suitable for PTR. According to a retrospective study, age does not cause senior gastric cancer patients to refuse surgery.^24^ However, the perspective must be applied with caution in mGC patients because the benefit of PTR must be evaluated when mGC patients are associated with aging‐related physiologic changes (e.g., decreasing organ function, pharmacokinetic and pharmacodynamic variability, and worsening functional status).^25^ Therefore, our study concluded that age should be specifically analyzed in conjunction with the total score of the nomogram. In our study, senior mGC patients (≥65 years) were more likely to be declared non‐beneficial candidates for PTR. In clinical practice, the prognosis of the histological type of signet‐ring cell is worse than that of non‐signet‐ring cell in the same advanced gastric cancer.^26^ Therefore, this study further raised the novel idea that mGC patients whose histologic type was signet‐ring cell had a lower PTR benefit than adenocarcinoma (exclude signet ring cell). According to our findings, this method identified the best candidate for primary site resection after a comprehensive assessment of the clinicopathological features of mGC patients. To the best of our knowledge, this is the first visualized nomogram on this topic.

It is worth noting that Sano et al classified mGC patients into four categories: (a) potential resectable metastasis, (b) borderline resectable metastasis, (c) incurable metastasis, and (d) unresectable metastasis.^23^ However, the dividing line between the four categories and how they relate to surgery remains vague. As a result, the patients in this study were separated into two groups: the surgery‐beneficial group and the surgery‐non‐beneficial group. By summing the scores, the nomogram quantifies the characteristics of mGC patients, estimating the boundary between PTR benefit and non‐benefit, and giving a reference for clinical evaluation. The actual benefit in this study referred to postoperative mGC patients who survived longer than the median OS and CSS time. In contrast, the benefit in the prognostic nomograms referred to a benefit recognized if the probability of benefit was >50%. The good predictive performance of the two predictive nomograms was proven by the validation of ROC curves, calibration curves, DCA, CIC, K‐M method, and log‐rank test.

After identifying patients who were candidates for surgery, assessing their prognosis after PTR was critical. Therefore, after establishing two predictive nomograms, we established two prognostic nomograms to assess the postoperative prognosis of mGC patients. Among them, sex, histologic type, grade, T stage, N stage, and chemotherapy were independent predictors to assess OS‐related prognostic nomogram, and histologic type, grade, T stage, N stage, and chemotherapy were independent predictors to assess CSS‐related prognostic nomogram.

Despite the strengths, there are several shortcomings in our study. Firstly, the SEER database does not contain complete clinicopathological data, such as smoking and drinking histories, previous underlying diseases, chemotherapeutic medications, treatment courses, targeted therapies, and immunological agents. Secondly, the study examined and screened patients with stage IV gastric cancer who were surgical candidates. Due to the limitations of the SEER database, the extent of metastasis has not been adequately characterized, including its nature, size, and sensitivity to pharmacological therapy. Thirdly, because this is a retrospective study, there is a need for more external multicenter prospective validation. Due to study and data limitations, using the SEER database is still a viable option for our research.

## 5 CONCLUSION

Our study provides validated predictive and prognostic nomograms. Predictive nomograms assist surgeons in identifying mGC patients who will benefit from PTR. Prognostic nomograms can help surgeons evaluate the prognosis of mGC patients after PTR. The novel models can help surgeons make better decisions and help specific patients.

## AUTHOR CONTRIBUTIONS

**Zhehong Li:** Data curation (equal); funding acquisition (equal); writing – original draft (equal); writing – review and editing (equal). **Honghong Zheng:** Methodology (equal); visualization (equal); writing – original draft (equal). **Ziming Zhao:** Writing – review and editing (equal). **Guanyang Chen:** Writing – review and editing (equal). **Zheng Wang:** Writing – review and editing (equal). **Buhe Amin:** Supervision (equal). **Nengwei Zhang:** Supervision (equal).

## FUNDING INFORMATION

We received no external funding for this study.

## CONFLICT OF INTEREST STATEMENT

The authors declare no potential conflicts of interest with respect to the research, authorship, and/or publication of this article.

## ETHICS APPROVAL AND CONSENT TO PARTICIPATE

The SEER data did not involve animal experiments, human specimens, or ethics‐related issues.

## Supporting information


Figure S1.

Figure S2.

Figure S3.

Figure S4.

Table S1‐S3.

## Data Availability

The datasets generated during and/or analyzed during the current study are available from the corresponding author on reasonable request.
