# Supplementary figures and images for: Identification of optimal primary tumor resection candidates for metastatic gastric cancer: Nomograms based on propensity score matching
Source: Cancer Med. 2023 Apr 25;12(12):13063–75. doi: 10.1002/cam4.5983 (PMC10315800; doi:10.1002/cam4.5983)

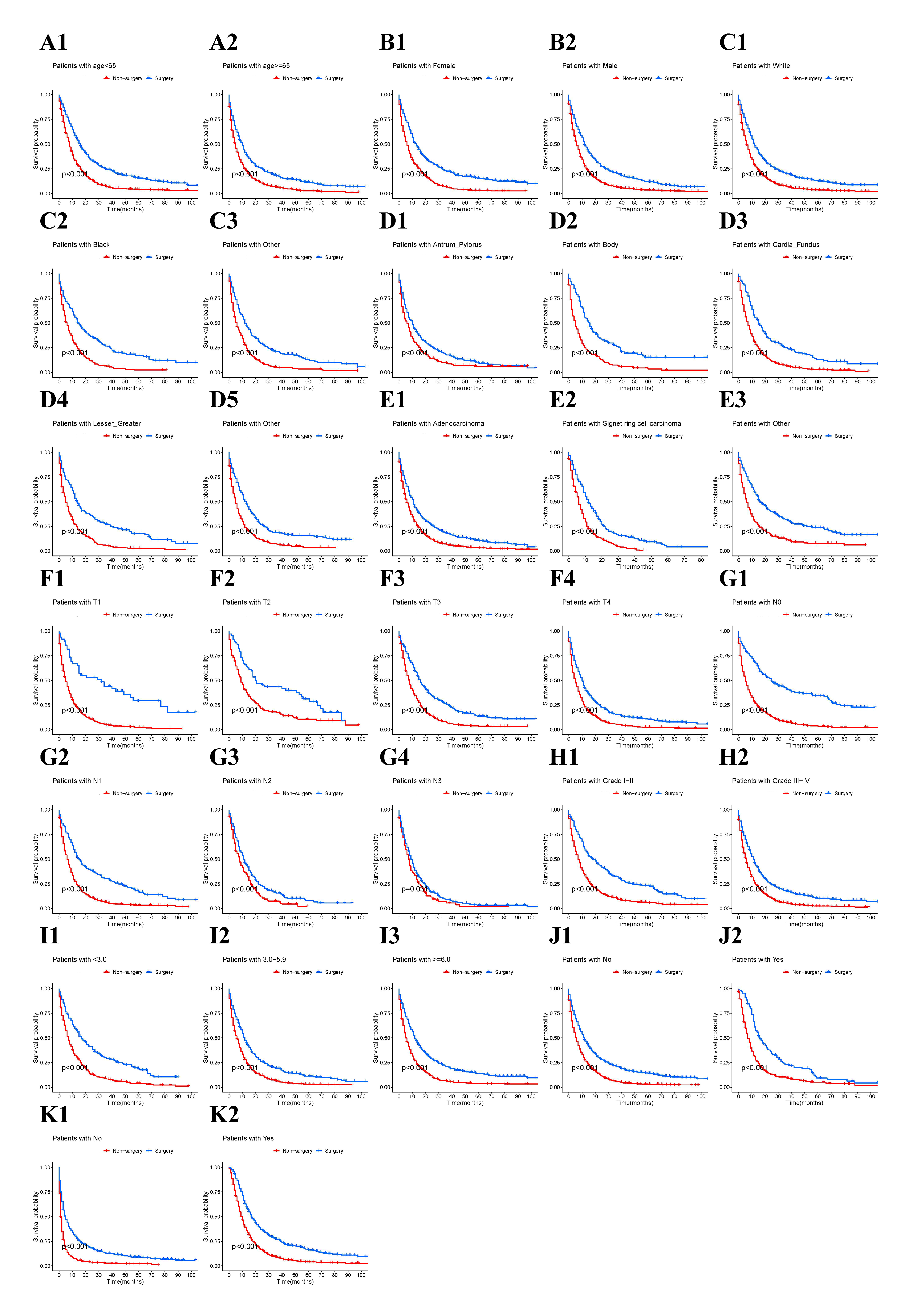

Supplement: Supplementary file 1 — Figure S1. [file CAM4-12-13063-s003.tif]

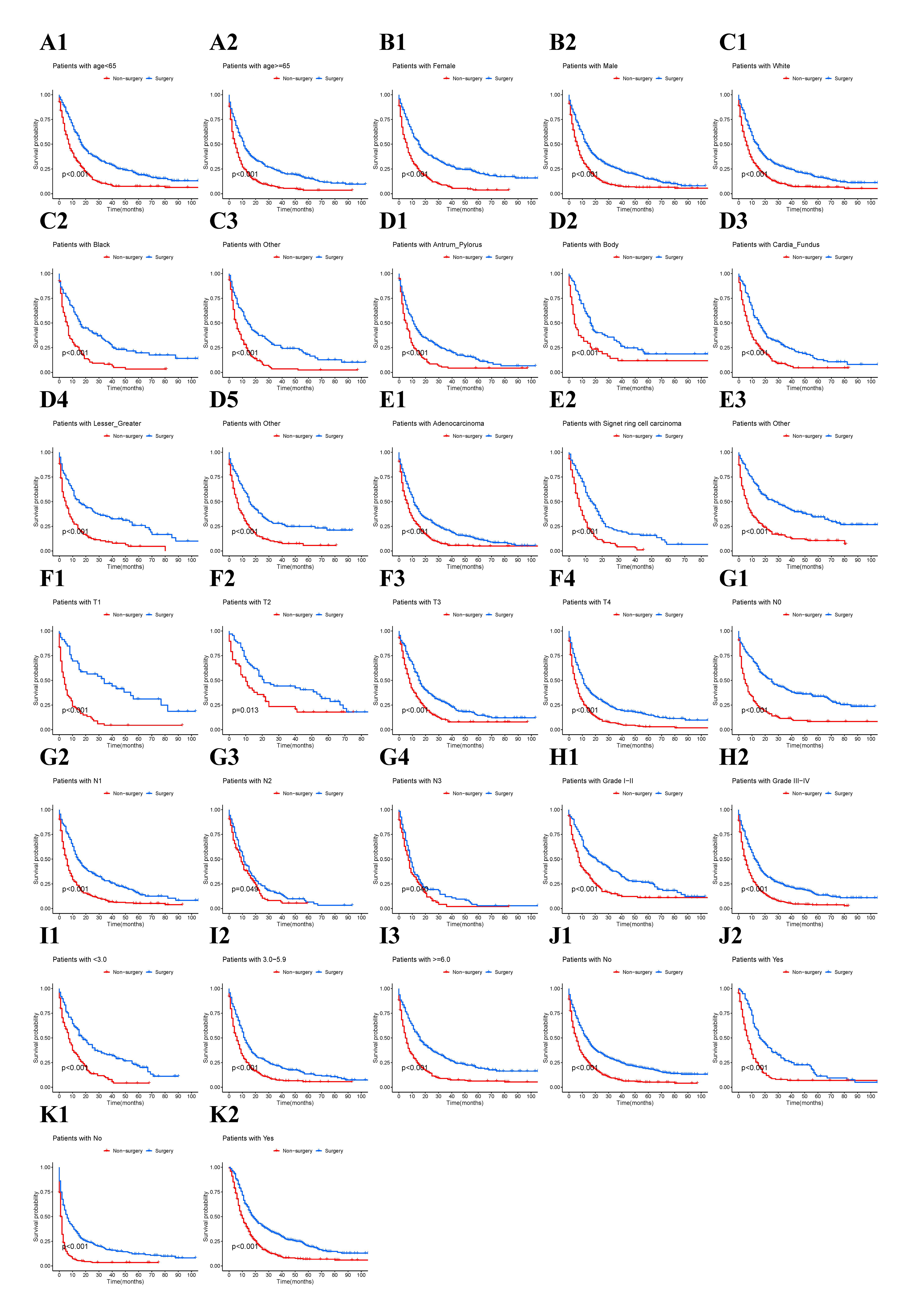

Supplement: Supplementary file 2 — Figure S2. [file CAM4-12-13063-s001.tif]

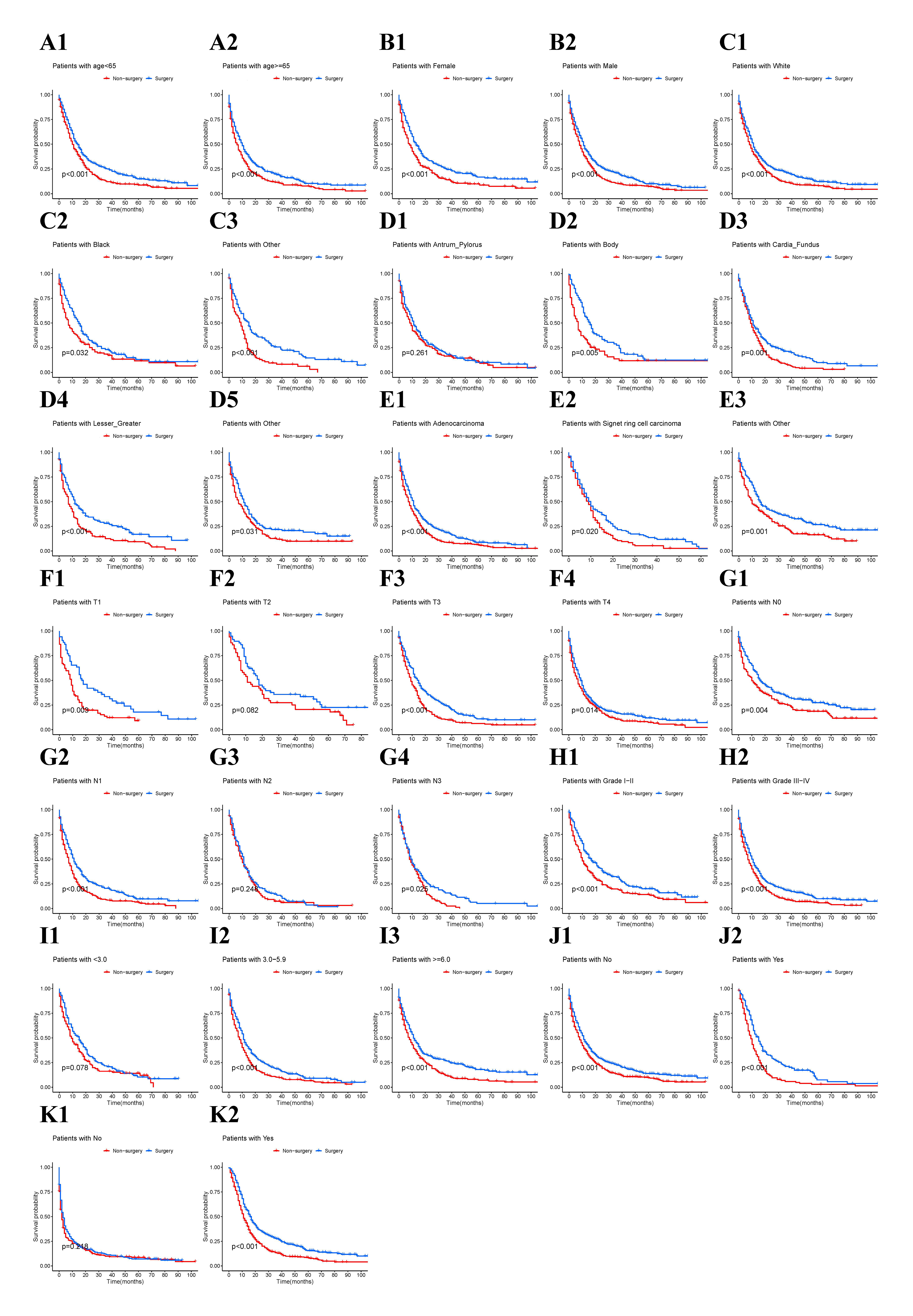

Supplement: Supplementary file 3 — Figure S3. [file CAM4-12-13063-s004.tif]

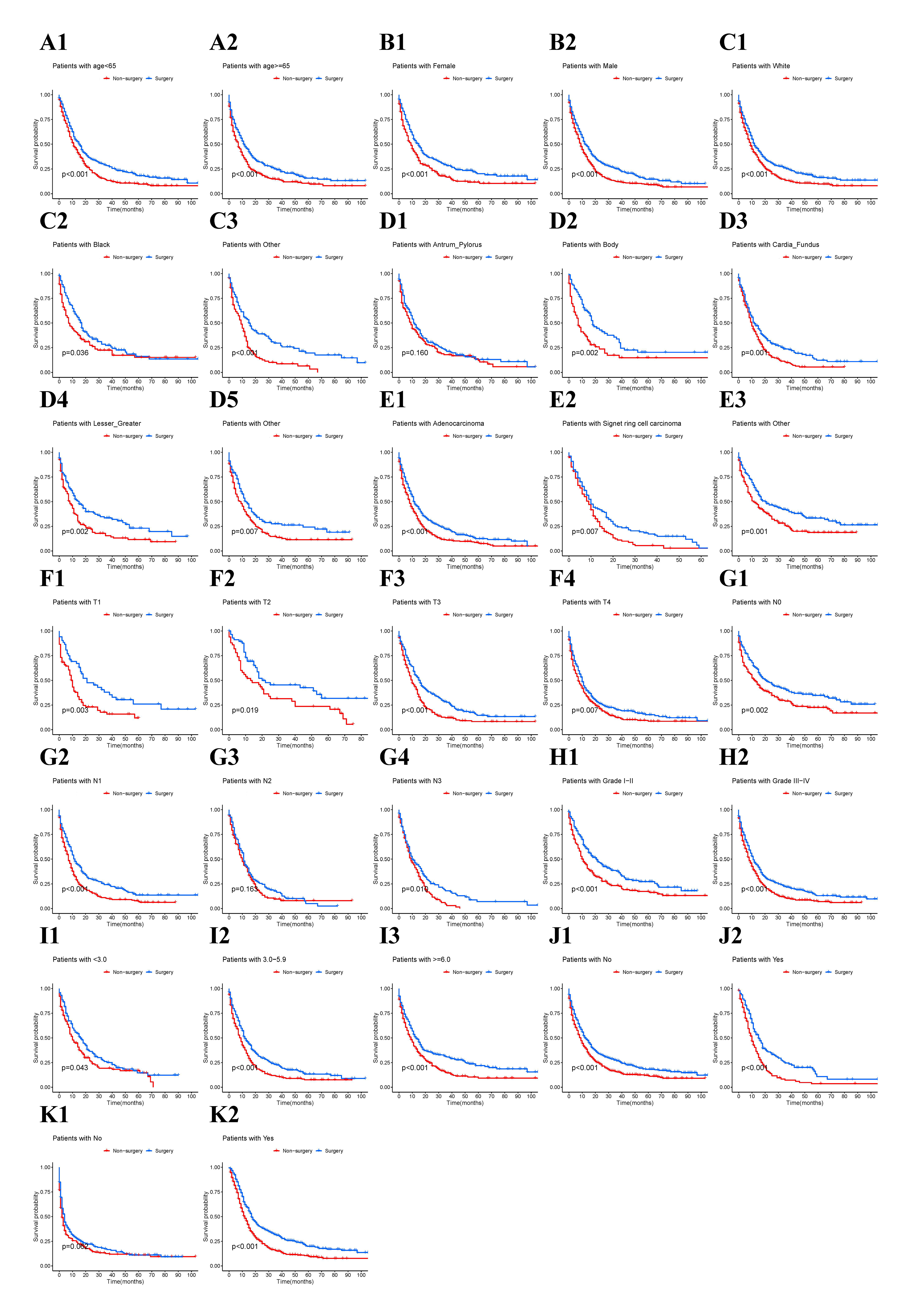

Supplement: Supplementary file 4 — Figure S4. [file CAM4-12-13063-s002.tif]
