# Supplementary material for: Identification of optimal primary tumor resection candidates for metastatic gastric cancer: Nomograms based on propensity score matching
Source: Cancer Med. 2023 Apr 25;12(12):13063–75. doi: 10.1002/cam4.5983 (PMC10315800; doi:10.1002/cam4.5983)
Supplement: Supplementary file 5 — Table S1‐S3. [file CAM4-12-13063-s005.docx]

**Supplementary Tables**

| Supplementary table 1. Clinical characteristics of the training and validation sets after PSM | | | |
| --- | --- | --- | --- |
| Variable | Training set  (N=528), n(%) | Validation set  (N=224), n(%) | *P* |
| Age (years) |  |  | 0.413 |
| ＜65 | 260(49.24) | 103(45.98) |  |
| ≥65 | 268(50.76) | 121(54.02) |  |
| Gender |  |  | 0.681 |
| Female | 192(36.36) | 85(37.95) |  |
| Male | 336(63.64) | 139(62.05) |  |
| Race |  |  | 0.890 |
| White | 358(67.80) | 155(69.20) |  |
| Black | 85(16.10) | 36(16.07) |  |
| Other | 85(16.10) | 33(14.73) |  |
| Histologic type |  |  | 0.578 |
| Adenocarcinoma  (exclude signet ring cell) | 296(56.06) | 125(55.80) |  |
| Signet ring cell | 89(16.86) | 32(14.29) |  |
| Other | 143(27.08) | 67(29.91) |  |
| Tumor site |  |  | 0.926 |
| Cardia and Fundus | 126(23.86) | 58(25.89) |  |
| Body | 65(12.31) | 29(12.95) |  |
| Antrum and pylorus | 170(32.20) | 71(31.70) |  |
| Lesser and greater | 74(14.02) | 32(14.29) |  |
| Other | 93(17.61) | 34(15.18) |  |
| Tumor size (cm) |  |  | 0.990 |
| ＜3.0 | 99(18.75) | 41(18.30) |  |
| 3.0-5.9 | 213(40.34) | 91(40.63) |  |
| ≥6.0 | 216(40.91) | 92(41.07) |  |
| Grade |  |  | 0.627 |
| Grade I-II | 151(28.60) | 68(30.36) |  |
| Grade III-IV | 377(71.40) | 156(69.64) |  |
| T stage |  |  | 0.935 |
| T1 | 32(6.06) | 16(7.14) |  |
| T2 | 47(8.90) | 18(8.04) |  |
| T3 | 215(40.72) | 91(40.63) |  |
| T4 | 234(44.32) | 99(44.20) |  |
| N stage |  |  | 0.315 |
| N0 | 171(32.39) | 67(29.91) |  |
| N1 | 149(28.22) | 76(33.93) |  |
| N2 | 114(21.59) | 50(22.32) |  |
| N3 | 94(17.80) | 31(13.84) |  |
| Radiotherapy |  |  | 0.279 |
| No | 428(81.06) | 189(84.38) |  |
| Yes | 100(18.94) | 35(15.63) |  |
| Chemotherapy |  |  | 0.319 |
| No | 178(33.71) | 84(37.50) |  |
| Yes | 350(66.29) | 140(62.50) |  |

| Supplementary table 2. Multivariate Logistics analysis for OS and CSS among PSM population | | | | |
| --- | --- | --- | --- | --- |
| Variables | OS | | CSS | |
|  | OR (95% CI) | *P* | OR (95% CI) | *P* |
| Age (years) |  |  |  |  |
| ＜65 | Reference |  | Reference |  |
| ≥65 | 0.464(0.288-0.750) | 0.002 | 0.596(0.369-0.964) | 0.035 |
| Gender |  |  |  |  |
| Female | Reference |  | Reference |  |
| Male | 0.745(0.453-1.225) | 0.246 | 0.896(0.546-1.470) | 0.663 |
| Race |  |  |  |  |
| White | Reference |  | Reference |  |
| Black | 1.417(0.708-2.838) | 0.325 | 1.610(0.793-3.269) | 0.188 |
| Other | 1.132(0.599-2.142) | 0.702 | 1.133(0.601-2.135) | 0.700 |
| Histologic type |  |  |  |  |
| Adenocarcinoma  (exclude signet ring cell) | Reference |  | Reference |  |
| Signet ring cell | 1.764(0.892-3.488) | 0.103 | 1.645(0.840-3.224) | 0.147 |
| Other | 2.136(1.177-3.877) | 0.013 | 2.171(1.182-3.988) | 0.012 |
| Tumor site |  |  |  |  |
| Cardia and Fundus | Reference |  | Reference |  |
| Body | 1.091(0.453-2.627) | 0.846 | 0.970(0.403-2.333) | 0.945 |
| Antrum and pylorus | 1.003(0.514-1.956) | 0.993 | 0.819(0.421-1.594) | 0.557 |
| Lesser and greater | 1.79(0.755-4.240) | 0.186 | 1.256(0.538-2.936) | 0.598 |
| Other | 1.082(0.494-2.370) | 0.845 | 1.310(0.585-2.934) | 0.511 |
| Tumor size (cm) |  |  |  |  |
| ＜3.0 | Reference |  | Reference |  |
| 3.0-5.9 | 1.635(0.843-3.172) | 0.146 | 1.330(0.682-2.592) | 0.402 |
| ≥6.0 | 1.294(0.659-2.541) | 0.454 | 1.223(0.619-2.414) | 0.563 |
| Grade |  |  |  |  |
| Grade I-II | Reference |  | Reference |  |
| Grade III-IV | 0.379(0.214-0.671) | 0.001 | 0.471(0.267-0.829) | 0.009 |
| T stage |  |  |  |  |
| T1 | Reference |  | Reference |  |
| T2 | 0.906(0.233-3.529) | 0.887 | 1.037(0.248-4.334) | 0.960 |
| T3 | 0.397(0.126-1.245) | 0.113 | 0.366(0.113-1.184) | 0.093 |
| T4 | 0.302(0.095-0.957) | 0.042 | 0.317(0.097-1.037) | 0.057 |
| N stage |  |  |  |  |
| N0 | Reference |  | Reference |  |
| N1 | 0.586(0.312-1.099) | 0.096 | 0.611(0.324-1.152) | 0.128 |
| N2 | 0.657(0.330-1.306) | 0.231 | 0.594(0.298-1.181) | 0.137 |
| N3 | 0.535(0.261-1.097) | 0.088 | 0.504(0.246-1.033) | 0.061 |
| Radiotherapy |  |  |  |  |
| No | Reference |  | Reference |  |
| Yes | 1.263(0.642-2.487) | 0.499 | 1.282(0.644-2.554) | 0.479 |
| Chemotherapy |  |  |  |  |
| No | Reference |  | Reference |  |
| Yes | 9.554(5.713-15.977) | ＜0.001 | 6.141(3.654-10.322) | ＜0.001 |

OS, overall survival; CSS, cancer-specific survival; PSM, propensity score matching; OR, odds ratio; CI, confidence interval.

| Supplementary table 3. Multivariate Cox analysis for OS and CSS among PSM surgery population | | | | |
| --- | --- | --- | --- | --- |
| Variables | OS | | CSS | |
|  | HR (95% CI) | *P* | HR (95% CI) | *P* |
| Age (years) |  |  |  |  |
| ＜65 | Reference |  | Reference |  |
| ≥65 | 1.209(0.981-1.491) | 0.075 | 1.137(0.910-1.421) | 0.258 |
| Gender |  |  |  |  |
| Female | Reference |  | Reference |  |
| Male | 1.272(1.024-1.580) | 0.029 | 1.232(0.978-1.552) | 0.076 |
| Race |  |  |  |  |
| White | Reference |  | Reference |  |
| Black | 0.992(0.738-1.332) | 0.955 | 1.041(0.760-1.426) | 0.188 |
| Other | 0.882(0.667-1.168) | 0.382 | 0.877(0.650-1.182) | 0.387 |
| Histologic type |  |  |  |  |
| Adenocarcinoma  (exclude signet ring cell) | Reference |  | Reference |  |
| Signet ring cell | 1.052(0.792-1.397) | 0.728 | 1.102(0.818-1.485) | 0.522 |
| Other | 0.699(0.536-0.913) | 0.008 | 0.689(0.517-0.918) | 0.011 |
| Tumor site |  |  |  |  |
| Cardia and Fundus | Reference |  | Reference |  |
| Body | 0.876(0.606-1.266) | 0.481 | 0.885(0.600-1.304) | 0.537 |
| Antrum and pylorus | 0.869(0.652-1.157) | 0.336 | 0.891(0.659-1.206) | 0.455 |
| Lesser and greater | 0.731(0.506-1.054) | 0.094 | 0.672(0.450-1.004) | 0.052 |
| Other | 0.769(0.546-1.083) | 0.133 | 0.695(0.479-1.007) | 0.054 |
| Tumor size (cm) |  |  |  |  |
| ＜3.0 | Reference |  | Reference |  |
| 3.0-5.9 | 1.051(0.784-1.410) | 0.737 | 1.035(0.757-1.415) | 0.830 |
| ≥6.0 | 0.951(0.701-1.291) | 0.749 | 0.899(0.650-1.243) | 0.520 |
| Grade |  |  |  |  |
| Grade I-II | Reference |  | Reference |  |
| Grade III-IV | 1.362(1.069-1.735) | 0.012 | 1.369(1.056-1.776) | 0.018 |
| T stage |  |  |  |  |
| T1 | Reference |  | Reference |  |
| T2 | 1.163(0.684-1.976) | 0.578 | 1.223(0.662-2.261) | 0.521 |
| T3 | 1.328(0.842-2.094) | 0.222 | 1.749(1.038-2.948) | 0.036 |
| T4 | 1.718(1.075-2.747) | 0.024 | 2.157(1.261-3.689) | 0.005 |
| N stage |  |  |  |  |
| N0 | Reference |  | Reference |  |
| N1 | 1.395(1.053-1.849) | 0.02 | 1.455(1.073-1.975) | 0.016 |
| N2 | 1.756(1.290-2.390) | ＜0.001 | 1.896(1.362-2.640) | ＜0.001 |
| N3 | 1.741(1.260-2.406) | 0.001 | 1.827(1.293-2.582) | 0.001 |
| Radiotherapy |  |  |  |  |
| No | Reference |  | Reference |  |
| Yes | 0.921(0.705-1.202) | 0.544 | 0.854(0.642-1.135) | 0.276 |
| Chemotherapy |  |  |  |  |
| No | Reference |  | Reference |  |
| Yes | 0.594(0.960-0.619) | ＜0.001 | 0.503(0.396-0.639) | ＜0.001 |

OS, overall survival; CSS, cancer-specific survival; PSM, propensity score matching; HR, hazard ratio; CI, confidence interval.
